# MEK inhibition reduces glial scar formation and promotes the recovery of sensorimotor function in rats following spinal cord injury

**DOI:** 10.3892/etm.2013.1371

**Published:** 2013-10-29

**Authors:** BIN LIN, YANG XU, BI ZHANG, YONG HE, YUN YAN, MING-CHANG HE

**Affiliations:** Department of Orthopaedics, The 175th Hospital of PLA, Southeast Hospital of Xiamen University, Zhangzhou, Fujian 363000, P.R. China

**Keywords:** extracellular signal-regulated kinases, glial fibrillary acidic protein, spinal cord injury, glial scar, vimentin

## Abstract

The aim of this study was to investigate the effect of U0126 on the formation of glial scars following spinal cord injury (SCI) in a rat model. Ninety adult female Sprague-Dawley rats were divided randomly into sham injury (group I), SCI (group II) and U0126 treatment (group III) groups, and functional outcome was observed during the 4 weeks following the injury. The P1 and N1 latencies and P1-N1 amplitudes of somatosensory-evoked potentials (SEPs) were collected one day prior to surgery, on the day of surgery and 14 and 28 days postoperatively. The expression levels of glial fibrillary acidic protein (GFAP) and vimentin (Vim) were assessed 14 and 28 days post-injury. Treatment with U0126 significantly increased locomotor function from the second week until 4 weeks post-SCI. At 14 and 28 days subsequent to the injury, the number of cells that were positive for GFAP expression in the U0126-treated group was significantly reduced and the GFAP-positive cells were observed to be smaller, with a reduced prominence and pale staining. Moreover, the area of glial scarring was smaller compared with that of the SCI controls. Inhibitors of MEK may reduce glial scar formation by suppressing the proliferation of astrocytes, and may improve hindlimb motor function.

## Introduction

Spinal cord injury (SCI) occurs predominantly in young people as a result of traffic or sports-related accidents and leads to severe neurological deficits, such as paraplegia and quadriplegia. SCI is usually accompanied by the formation of cystic cavities surrounded by glial scars, which severely impede the regeneration of severed axons (1). The poor recovery of the central nervous system, a delicate tissue that is unable to tolerate toxic conditions, is generally attributed to the hostile local environment created at the trauma site. Two major barriers to repair that have been identified include the local inflammatory response, acknowledged for its neurotoxic potential, and the creation of glial scars, which has been demonstrated to impair regeneration (1–3). Glial scars are composed of extracellular matrices and various types of cells; astrocytes, in particular, are important in glial scar formation. Astrocytes upregulate the expression of glial fibrillary acidic protein (GFAP) (4,5), as well as re-expressing vimentin (Vim) and secreting a number of extracellular matrix proteins following SCI. In particular, chondroitin sulfate proteoglycans (CSPGs) are considered to be the primary component of extracellular matrix proteins (6–8). Although reactive astrocytes may exert a number of beneficial effects by regulating local immune responses and promoting tissue repair (9,10), results from numerous studies have indicated that glial scarring is one of the major factors hindering spontaneous axonal regeneration, and that the suppression of glial scarring may reduce tissue damage and improve morphological/functional recovery (11–14). For these reasons, interventions designed to attenuate astrogliosis represent valuable therapeutic agents for the management of SCI.

The activation of extracellular signal-regulated kinase (ERK) by mitogen-activated protein kinase (MAPK)/ERK (MEK) through phosphorylation is an important step in signal transduction for cell processes that transfer chemical signals from the cell surface to the nucleus (15,16). It has been demonstrated that the inhibition of the ERK signaling pathway provided neuroprotection in cell models of mechanical trauma (17). Furthermore, Lu *et al*(18) revealed that in a rat model of spinal cord injury, MEK/ERK inhibition reduced microglial activation, cytokine production and inflammatory cell infiltration. The MEK inhibitor U0126 inhibited ERK phosphorylation and the migration of astrocytes across a wound, and the migration of human astrocytes following injury has been indicated to be partly initiated by the activation of the MEK/ERK signaling pathway (19). Inhibiting ERK phosphorylation with U0126 has been shown to significantly attenuate apoptotic neuronal loss and improve neurological function (20). The MEK/ERK signal transduction pathway may be critical in the formation of the microenvironment in SCI. However, it has not been fully investigated whether inhibitors of MEK are able to regulate glial scar formation and improve functional recovery following SCI.

In the present study, the ability of an inhibitor of MEK1/2 to affect astrocytic scar formation following SCI was examined. The results in this study indicated that U0126 inhibited astrocyte proliferation, as well as the expression of CSPGs, *in vivo*. In addition, an improvement in hindlimb motor function was observed. Therefore, this study demonstrated a therapeutic potential for inhibitors of MEK in the regulation of glial scar formation and the promotion of axonal regeneration and functional recovery following SCI.

## Materials and methods

### Animals and surgical procedures

All experimental procedures were performed in accordance with protocols approved by the Governmental Animal Care Committee of the Medical College of Xiamen University (Zhangzhou, China) and conformed to the National Institute of Health guidelines on the ethical use of animals. Prior to surgery, the animals were anesthetized with chloral hydrate *(*400 mg/kg, intraperitoneally; Beyotime Institute of Biotechnology, Haimen, China). During surgery, the rats were placed on a warming pad to maintain a body temperature of 37.0±0.5°C. Following injury, the animals were returned to individual cages with sufficient water and food and were then treated with an intramuscular injection of penicillin (The 175th Hospital of PLA, Zhangzhou, China) at a dose of 200,000 U/day, for three days.

Adult female Sprague Dawley rats (weight, 240–260 g) were randomly assigned into three experimental groups: sham injury (group I, n=30), SCI (group II, n=30) and U0126 treatment (group III, n=30). U0126 was obtained from Beyotime Institute of Biotechnology. The rats were obtained from Experimental Animal Center of Xiamen University. The traumatic SCI model was established by the weight-drop technique, as described in a previous study (21). Briefly, following anesthetization, a T12 spinal laminectomy was conducted to expose the spinal cord and a moderate-intensity weight-drop (10 g × 7.0 cm) was performed using an impactor with a diameter of 2.5 mm (Xiamen University). The rats in the sham surgery group underwent a similar experimental procedure to that used for SCI induction, with the exception of the weight-drop step; saline was administered to rats in the sham injury and SCI groups through pumps, as described in the following section.

Bladders were manually pressed twice daily until spontaneous voiding occurred and food and water were freely accessible at a lowered height in the cages. U0126 was administered to the rats in group III (n=30) at a dose of 20 μg/day by intraperitoneal (ip) injection within 1 h subsequent to SCI until 9 days post-injury. The experimental control rats (group II, n=30) were treated with ip injections of saline instead of U0126. The normal control rats without SCI (group I, n=30) were maintained throughout the experiment.

### Behavioral assessments

The rats were tested for locomotor deficits at 1, 3, 5, 7 and 14 days (n=30, per group) and 21 and 28 days (n=15, per group) subsequent to SCI with the open field locomotor test, developed by Basso *et al*(22). This Basso, Beattie and Bresnahan (BBB) locomotor rating scale was conducted by two observers, who were blinded to the experimental procedures of each rat. The BBB rating scale is a 21-point system based on operationally defined behavioral features, which follow the recovery progression from complete paralysis to normal locomotion. The rating scale ranges from 0 to 21, with a score of 0 indicating complete hind limb paralysis and a score of 21 denoting completely normal locomotor function.

### Somatosensory-evoked potentials (SEPs)

The rats were anesthetized and 1 min later an incision was made along the midline of the back. The cranium bone was cleaned by removing the tissue under the skin. A standard dental drill was used to drill five burr holes into the exposed area of the cranium. Four holes were located on the somatosensory cortex corresponding to the hind and forelimbs in each hemisphere. On each hemisphere, the forelimb recording sites were located 0.2 mm posterior to the bregma and 3.8 mm laterally from the bregma, and the hindlimb recording sites were located 2.5 mm posterior to the bregma, and 2.8 mm laterally from the bregma. A fifth hole, drilled on the right frontal bone and situated 2 mm from the sagittal and coronal sutures, served as the intracranial reference. Transcranial screw electrodes were then screwed into the holes, such that they made very light contact with the dura mater. The distal end of each electrode was inserted into one of the slots of an electrode pedestal. Sub-dermal needle electrode pairs (MedCom Asia, Inc., Guangzhou, China) were used to electrically stimulate the tibial nerves of the left and right hind limbs. An isolated constant current stimulator (Shanghai Haishen Medical Electronic Instrument Co., Ltd., Shanghai, China) was used for the electrical stimulation of the limbs. A Microsoft Windows-based personal computer was interfaced with the stimulator and a neurological monitoring system (Model M-800; MedCom Asia, Inc.) was used to set the stimulation parameters and trigger the stimulator. The values of P1 latency, N1 latency and P1-N1 amplitude were collected one day prior to surgery, on the day of surgery and 14 and 28 days postoperatively, and were subject to statistical comparisons.

### Tissue processing, staining and histopathology

On days 14 and 28 post-injury, three rats from each group were sacrificed for immunohistological staining. Sections of the spinal cords encompassing the injured sites were dissected and fixed by immersion in 4% formaldehyde for 24 h, and cryoprotected in 10% sucrose at 4°C until they sank. The spinal cords were then embedded in optimum cutting temperature (OCT) compound (Beyotime Institute of Biotechnology), frozen and cut into 3-μm cryostat sections in the horizontal plane. The tissue sections were stained with a Hematoxylin and Eosin (H&E) Staining kit (Beyotime Institute of Biotechnology) to assess the morphology of the injury site. Eight to ten sections were stained for GFAP or Vim to identify reactive astrocytes. The immunohistochemical staining was performed using a Histonstain^®^-Plus kit (Invitrogen Life Technologies, Carlsbad, CA, USA), in accordance with the manufacturer’s instructions. The images were captured using a FV 300 confocal microscope (Olympus, Tokyo, Japan).

### Statistical analysis

Statistical analysis was performed with the Statistical Package for Social Sciences (SPSS) version 13.0 for Windows (SPSS, Inc., Chicago, IL, USA). The Mann-Whitney U test was used to compare the BBB score and positive cell count, and SEP was analyzed using one-way analysis of variance (ANOVA). P<0.05 was considered to indicate a statistically significant difference. Data are presented as the mean ± standard error of the mean (SEM).

## Results

### Behavioral test

The BBB locomotor scale was used to evaluate all rats prior to the electrophysiological evaluation. Table I shows the mean BBB scores for the three groups over the time-course of the experiment. All rats were healthy prior to surgery and exhibited BBB scores of 21 (data not shown). In the sham injury group, there was no significant difference between the hindlimb movement scores prior to and following SCI and the movement appeared normal throughout the observation period (BBB 21 points). The SCI and U0126-treated groups showed motor function improvements one day subsequent to injury, although the U0126-treated group exhibited better total recovery than the control group by day 28, scoring an average of 16.50±1.08 points. The control group scored 12.00±1.70 points on day 28 (P<0.05). A score of 10 indicated only occasional weight-supported plantar steps and no front-hind limb coordination, while a score of 14 meant consistent weight-supported plantar steps and consistent front-hind limb coordination (22). Thus, it was noteworthy that the U0126 group, and not the control group, exceeded this threshold.

### SEPs

Tables II and III show the latency and amplitude of the SEPs, respectively. The day subsequent to injury, the mean SEP latency was observed to be notably extended, while the amplitudes were observed to have decreased markedly in group II and the U0126-treated group. In the U0126-treated group 14 days after injury, the mean SEP latency had decreased and the SEP amplitude had increased compared with the corresponding values on the day subsequent to injury. The difference between the these two groups was statistically significant (P<0.05). The mean SEP amplitudes of the U0126-treated group were consistently higher than those of group II. The difference between the two groups was revealed to be statistically significant for the majority of the weekly recordings (P<0.05). As Table II shows, the SEP latencies were shorter in the U0126-treated group than in group II and the difference between the SEP latencies of these groups was statistically significant on days 14 and 28 post-injury (P<0.05).

### Histological assessments

#### Visual study

Following injury, diffuse hyperemia and edema were immediately observed in the dorsal region of the spinal cord in groups II and III; at 14 and 28 days post-SCI, scar formation was visible on the outside of the endorhachis of the lesion region and notable conglutination with the endorhachis was apparent. In addition, the spinal cord was atrophic, with a reduced diameter. In group I, at 14 and 28 days post-SCI, a small amount of scar formation was observed in the outside of the endorhachis of the lesion region and conglutination with the endorhachis was apparent. This may have been due to the stimulus of the surgery when opening the vertebral plate. However, there was no evident edema in the spinal cord and the posterior central blood vessel and the structure of the spinal cord were clearly visible.

#### H&E staining

**Figure 1:**
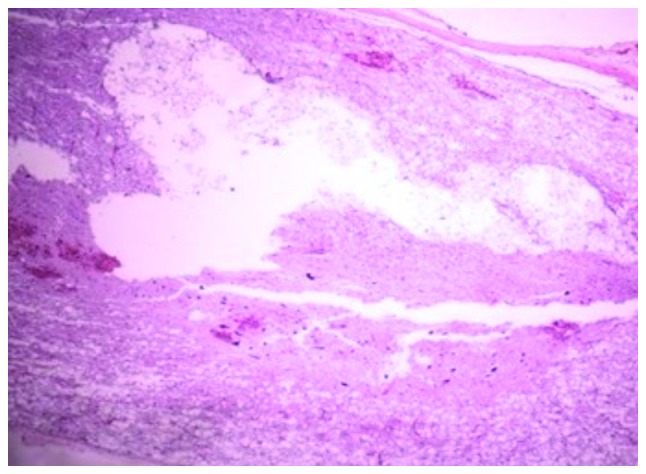
Twenty-eight days subsequent to spinal cord injury (SCI), hematoxylin and eosin (H&E) staining in group II showed that the spinal cord gray and white matter hemorrhaging had been absorbed and the neurons had dissolved and liquefied, forming a large number of vacuolar structures. The inflammatory infiltration was reduced and glial scar formation was apparent, with a large number of surrounding glial cells. Magnification, ×100.

**Figure 2:**
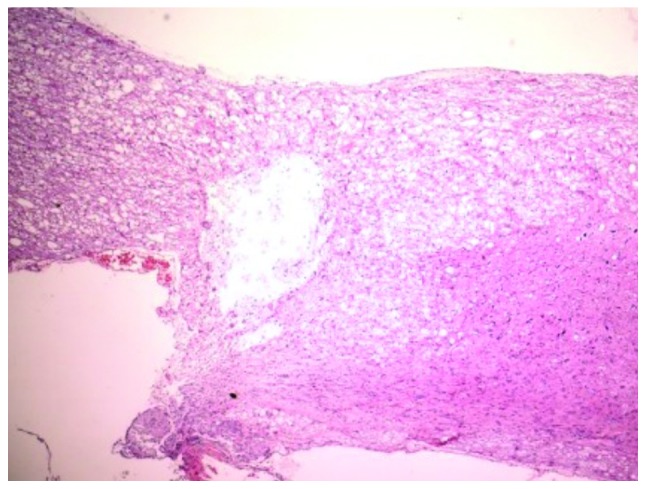
Twenty-eight days subsequent to spinal cord injury (SCI), hematoxylin and eosin (H&E) staining in the U0126-treated group showed that the infiltration of inflammatory cells was reduced and a scar had formed, although the size of the scar was smaller compared with that in Group II. Magnification, ×100.

At 14 days post-SCI, H&E staining in group II showed that a little hemorrhagic focus was apparent in the gray and white matter of the spinal cord and that the structure of the spinal cord was badly destroyed, with neurons dissolved and liquefied in the gray matter. This caused a large liquefied and necrotic area, forming a cystic space. In addition, a large number of swollen axons and vacuoles were observed in the white matter and the nerve fibers were disorganized. At 28 days, the hemorrhagic focus in the gray and white matter was almost completely absorbed and the structure of the spinal cord was destroyed further, with neurons dissolved and liquefied in the gray matter, forming a large number of vacuolar structures. In addition, there was a reduction in the inflammatory infiltration, and the formation of a pyknotic glial scar, surrounded by a large number of glial cells, was observed (Fig. 1). At 14 days post-SCI, the H&E staining in group III also showed that the structure of the spinal cord was destroyed, with the infiltration of inflammatory cells, dehydration and disintegration of the neurons, hyperplastic and hypertrophic gliocytes and the formation of cystic space. However, the degree and area of the damage was reduced compared with those in group II. At 28 days post-SCI, the H&E staining in group III showed that the infiltration of inflammatory cells was reduced and that the size of the formed scar was smaller than that in group II (Fig. 2). The H&E staining in group I at 14 and 28 days post-SCI showed that there were no evident changes in most of the structure of the spinal cord; the structure of the neurons was clearly visible, the outer limits of the gray and white matter were marked and there were no cystic spaces. However, a small amount of hemorrhaging was observed in the spinal cord of a few rats and there was a slight gathering of gliocytes. This may have been associated with a reduction in the stability of the spine and the subsequent injury of the spinal cord for the resection of the vertebral plate.

#### GFAP immunohistochemical results

**Figure 3:**
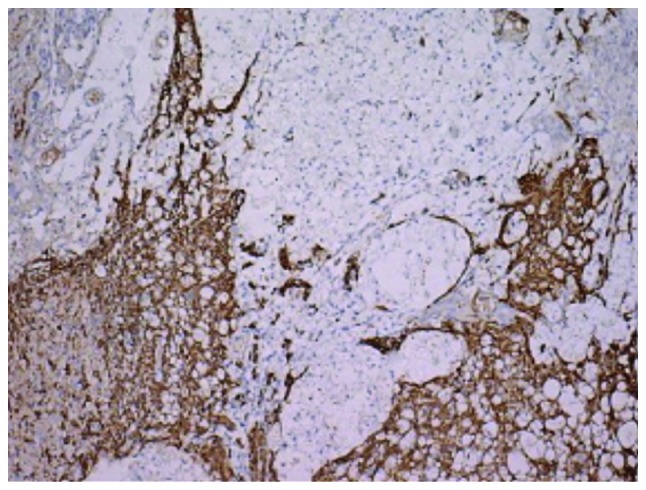
At 14 days post-spinal cord injury (SCI), the number of glial fibrillary acidic protein (GFAP)-positive cells was enhanced. Cells were deeply stained and showed hypertrophy and neurite extension. A number of positive cells surrounded the cystic cavity. Astrocyte proliferation and hypertrophy were also observed near the injury, although the extent of the proliferation was less than the injury area. Magnification, ×100.

**Figure 4:**
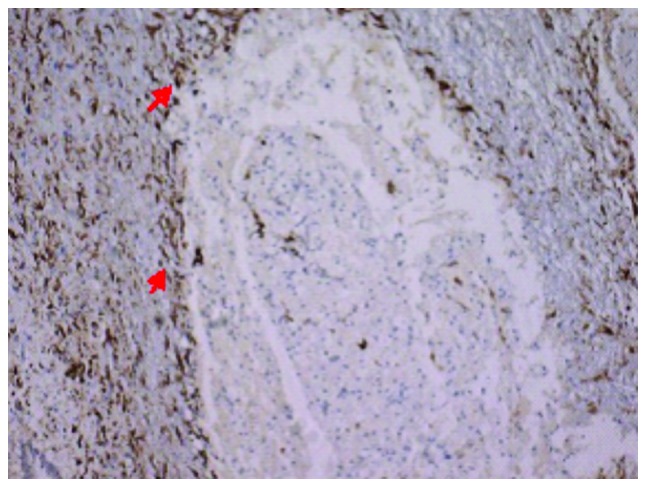
Compared with the injury at 14 days subsequent to the spinal cord injury (SCI), at 28 days post-SCI the number of glial fibrillary acidic protein (GFAP)-positive cells was significantly reduced. However, the prominence of the positive cells was thicker and longer, woven into reticulate structure and formed a dense glial scar. The arrows indicate the region of glial scar. Magnification, ×100.

**Figure 5:**
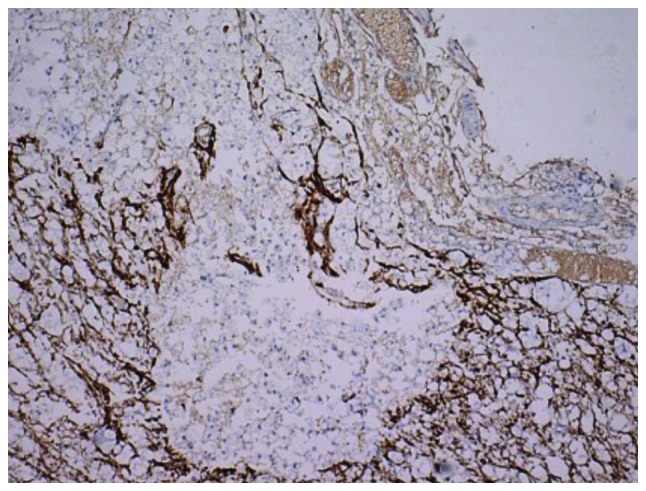
Expression of glial fibrillary acidic protein (GFAP)-positive cells in the U0126-treated group 14 days subsequent to spinal cord injury. Magnification, ×100.

**Figure 6:**
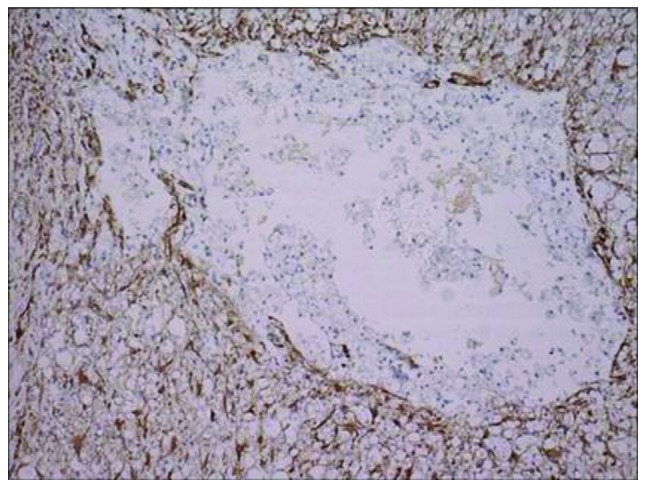
Twenty-eight days subsequent to spinal cord injury (SCI), the number of glial fibrillary acidic protein (GFAP)-positive cells in the U0126-treated group was significantly different compared with that in group II (P<0.05). The GFAP-positive cells were smaller in size, with a reduced prominence and pale staining. The area of glial scarring was also smaller. Magnification, ×100.

The numbers of GFAP-positive cells in each group on days 14 and 28 are listed in Table IV. In the GFAP immunohistochemical staining, the cytoplasm of the positive cells was brown and radially formed, spider-like projections were observed. At 14 and 28 days post-SCI, the staining in the sham injury group showed a small volume of positive cells, with a relatively sparse density. The nerve structure was visible. At 14 days post-SCI, the number of GFAP-positive cells in group II was increased. As shown in Fig. 3, the cells were deeply stained, showing hypertrophy and neurite extension. A number of positive cells surrounded the cystic cavity. Astrocyte proliferation and hypertrophy were also observed near the injury, although the extent of the proliferation was less than the injury area. At 28 days post-SCI, the number of GFAP-positive cells in group II had significantly reduced compared with that at 14 days post-SCI. However, the prominence of the positive cells was thicker and longer, woven into reticulate structure and formed a dense glial scar (Fig. 4). As Table IV shows, there were more GFAP-positive cells in group II than in the U0126-treated group and the difference between the two groups was statistically significant (P<0.05). As shown in Figs. 5 and 6, respectively, at 14 and 28 days post-SCI, the number of GFAP-positive cells in group III was significantly reduced and the GFAP-positive cells became smaller, with a reduced prominence and pale staining. In addition, the scope of the glial scar was smaller.

#### Vim immunohistochemical results

**Figure 7:**
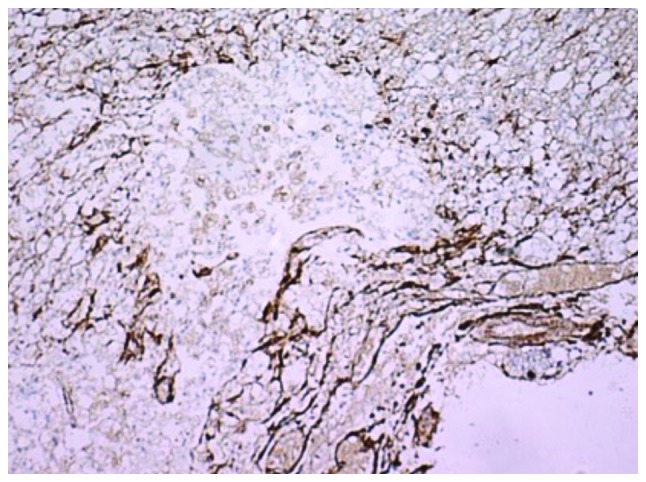
Following spinal cord injury (SCI), the vimentin (Vim) immunohistochemical staining showed that the cytoplasm of the positive cells contained brown particles and radially formed, spider-like projections were visible. Magnification, ×100.

**Figure 8:**
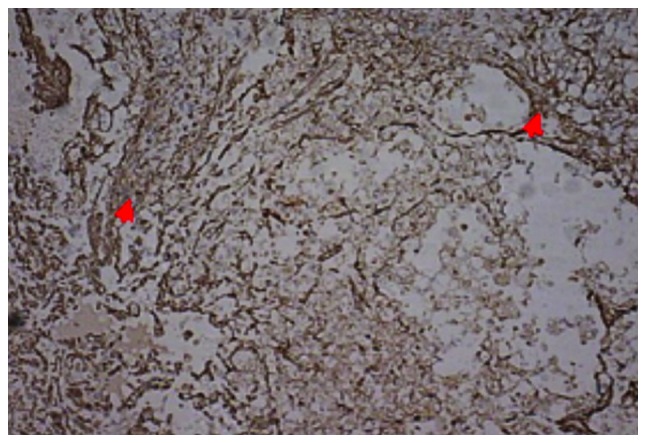
At 28 days subsequent to injury, the number of vimentin (Vim)-positive cells in the U0126-treated group was significantly decreased compared with the number in group II. The arrows indicate the region of glial scar. Magnification, ×100.

The number of the Vim-positive cells in each group on days 14 and 28 are listed in Table V. The Vim immunohistochemical staining revealed that there were no positive cells in group I, and that the structural integrity of the neurons was retained. In groups II and III, the Vim immunohistochemical staining showed that the cytoplasm of positive cells contained brown particles and radially formed, spider-like projections were observed. At 14 days subsequent to SCI, the number of Vim-positive cells in group II was increased and a number of positive cells were observed to surround the cystic cavity (Fig. 7). At 28 days subsequent to SCI, the number of Vim-positive cells in group III was significantly lower than that in group II (Fig. 8) and the difference between the two groups was statistically significant (P<0.05).

## Discussion

Traumatic SCI is a devastating ailment that leaves the majority patients with permanent neurological deficits. At present, the treatment for patients with SCI centers on the surgical stabilization of the initial injury to prevent further loss of neurological function, followed by aggressive rehabilitation. A previous study has demonstrated the ability of the nervous system to adapt to SCI from a functional (neuronal plasticity) and a structural (neuronal remodeling) standpoint (23). However, the formation of a glial scar following SCI has been suggested to create a microenvironment that is unfavorable for continued axonal regeneration and neurological recovery (24,25). Following SCI, astrocytes become hypertrophic, proliferate and show an overexpression of GFAP and the re-expression of Vim. Hypertrophic astrocytes are the major cellular component of the glial scar (1). Increasingly, studies have shown that microglial activation is one of the major causes of secondary damage subsequent to SCI, and that the suppression of microglial activation may reduce tissue damage and improve morphological/functional recovery (26,27). The data presented in this study have provided novel insights into the unusual role of MEK/ERK signaling in astrocyte activation.

The MAPK family includes ERK, p38MAPK and c-Jun N-terminal kinase (JNK) (28). The initiation of the ERK/MAPK cascade involves the activation of three kinases: Ras, Raf and MEK, and the ERK/MAPK pathway has traditionally been considered to be important in cell proliferation and differentiation (29–32). As mentioned previously, phosphorylated ERK may be expressed in neurons, microglia and astrocytes, with particularly persistent expression levels in astrocytes (33). This study aimed to explore whether the MEK/ERK signaling pathway participated in the process of glial scar formation. In this study, the MEK inhibitor U0126 was used to treat rats with SCI. Cells positive for GFAP and Vim, two markers used to identify reactive astrocytes (34), were detected as indicators of astrocyte proliferation. Following SCI, it was observed that there was an overexpression of GFAP and a re-expression of vimentin in the SCI group compared with the sham group. However, the GFAP and Vim expression was significantly reduced in the U0126 treatment group, and in this group, the volume of positive cells was reduced, the glial scar was smaller than that in the SCI group and the positive cell density was sparser. It has been demonstrated that the U0126 MEK inhibitor downregulates the expression of GFAP and Vim and that it may be possible to inhibit the formation of glial scars by reducing the proliferation of astrocytes. The results of the present study suggested that there is potential for Vim to be used as the indicator of gliosis following SCI, as Vim failed to be expressed in the sham injury group. This was consistent with a previous study (35).

It has been suggested that the MEK/ERK signaling pathway participates in the inflammatory reaction and the process of triggering the negative factors of SCI (18,36), which has been considered to involve cell apoptosis (37,38). By blocking this signaling pathway, the inflammatory reaction and apoptotic regeneration are inhibited and neurological recovery is stimulated. The current study demonstrates the potential benefits of treatment with MEK inhibitors following SCI and the effect of the treatment on the preservation of the ascending somatosensory pathway using SEP monitoring. The results showed improvements in SEP amplitudes lasting for several weeks. This was also accompanied by increased motor behavioral scores and histological preservation, indicative of neuroprotection. Importantly, early enhancements of SEP amplitudes in the U0126-treated rats indicated that the benefits lay in the preservation of somatosensory conductivity following injury.

We propose that the MEK/ERK signaling pathways may be involved in the formation of glial scars. Furthermore, this study has underlined the potential of U0126 for improving the functional outcome following SCI. Future studies are required to detail the pathophysiological events that activate the ERK/MAPK pathway and spinal cells, in order to advance the understanding of the role of ERK phosphorylation and spinal cells in the mechanisms underlying SCI.

## Figures and Tables

**Table I:** BBB score results of each group at different times.

Time-point	Group I (score)	Group II (score)	Group III (score)
Day of injury (n=12)	21.00±0.00	00.00±0.00^a^	00.00±0.00^a^
1 day post-injury (n=12)	21.00±0.00	1.65±0.67^a^	1.45±0.89^a^
3 days post-injury (n=12)	21.00±0.00	3.50±0.69^a^	3.45±0.69^a^
5 days post-injury (n=12)	21.00±0.00	5.70±0.80^a^	5.20±1.15^a^
7 days post-injury (n=12)	21.00±0.00	7.80±0.76^a^	8.15±1.04^a^
14 days post-injury (n=12)	21.00±0.00	9.65±1.50^a^	13.70±1.26^a^,^b^
21 days post-injury (n=6)	21.00±0.00	10.40±1.51^a^	14.10±1.52^a^,^b^
28 days post-injury (n=6)	21.00±0.00	12.00±1.70^a^	16.50±1.08^a^,^b^

a Compared with group I, P<0.05;

b Compared with group II, P<0.05.

BBB, Basso, Beattie and Bresnahan; group I, sham injury; group II, spinal cord injury (SCI); group III, SCI with U0126 treatment.

**Table II:** SEP latency at different times in each group (msec; n=15).

Group	Before injury	1 day post-injury	14 days post-injury	28 days post-injury
Group I	13.54±0.39	13.62±0.39	13.60±0.45	13.58±0.45
Group II	13.57±0.46	23.36±0.36^a^	20.41±0.34^a^	17.43±0.44^a^
Group III	13.57±0.29	23.53±0.42^a^	19.72±0.43^a^,^b^	16.86±0.55^a^,^b^

Results are presented as the mean ± standard error of the mean.

a Significantly different compared with group I (P<0.05);

b significantly different compared with group II (P<0.05).

SEP, somatosensory-evoked potential; group I, sham injury; group II, spinal cord injury (SCI); group III, SCI with U0126 treatment.

**Table III:** SEP amplitude at different times in each group (μV; n=15).

Group	Before injury	1 day post-injury	14 days post-injury	28 days post-injury
Group I	5.98±0.22	6.02±0.25	6.02±0.42	5.99±0.30
Group II	5.97±0.25	1.71±0.28^a^	3.38±0.50^a^	3.79±0.41^a^
Group III	5.96±0.23	1.74±0.32^a^	3.84±0.33^a^,^b^	4.19±0.11^a^,^b^

Results are presented as the mean ± standard error of the mean.

a Significantly different compared with group I (P<0.05);

b significantly different compared with group II (P<0.05).

SEP, somatosensory-evoked potential; group I, sham injury; group II, spinal cord injury (SCI); group III, SCI with U0126 treatment.

**Table IV:** GFAP-positive cells at different times in each group (n=15).

Group	14 days post-injury (n)	28 days post-injury (n)
Group I	27.82±1.29	27.1±1.66
Group II	143.56±1.09^a^	110.68±9.41^a^
Group III	133.56±3.31^a^,^b^	102.44±6.93^a^,^b^

Results are presented as the mean ± standard error of the mean.

a Significantly different compared with group I (P<0.05);

b significantly different compared with group II (P<0.05).

GFAP, glial fibrillary acidic protein; group I, sham injury; group II, spinal cord injury (SCI); group III, SCI with U0126 treatment.

**Table V:** Vim-positive cells at different times in each group (n=15).

Group	14 days post-injury (n)	28 days post-injury (n)
Group I	0.00±0.00	0.00±0.00
Group II	93.82±4.48^a^	72.96±4.16^a^
Group III	89.32±6.50^a^,^b^	66.44±4.46^a^,^b^

Results are presented as the mean ± standard error of the mean.

a Significantly different compared with group I (P<0.05);

b significantly different compared with group II (P<0.05).

Vim, vimentin; group I, sham injury; group II, spinal cord injury (SCI); group III, SCI with U0126 treatment.
